# Valorization of *Carapa guianensis* By-Products: Extraction Optimization and Antimicrobial and Antioxidant Activity

**DOI:** 10.3390/foods15152641

**Published:** 2026-07-28

**Authors:** Vinicius Sidônio Vale Moraes, Gabriela Vieira Pantoja, José Aparecido Ferreira de Lima, Emídio Beraldo-Neto, Emanuelle da Silva Prudente, Johnatt Allan Rocha de Oliveira, Luiza Helena da Silva Martins, Lúcia de Fátima Henriques Lourenço, Daniel Carvalho Pimenta, Gustavo Guadagnucci Fontanari

**Affiliations:** 1 Programa de Pós-Graduação em Ciência e Tecnologia de Alimentos (PPGCTA), Universidade Federal do Pará (UFPA), Rua Augusto Corrêa, Belém 66075-900, Pará, Brazil; vinicius.moraes@itec.ufpa.br (V.S.V.M.); gabriela.pantoja@itec.ufpa.br (G.V.P.); johnatt@ufpa.br (J.A.R.d.O.); luiza.martins@ufra.edu.br (L.H.d.S.M.); luciahl@ufpa.br (L.d.F.H.L.); 2 Laboratorio de Bioquimica, Instituto Butantan, São Paulo 05503-900, São Paulo, Brazil; jose.aparecido@fundacaobutantan.org.br (J.A.F.d.L.); emidio.beraldo@butantan.gov.br (E.B.-N.); 3 Programa de Pós Graduação em Biotecnologia Aplicada a Agropecuária (PPGBAA), Belém 66077-830, Pará, Brazil; emanuelleprudente4@gmail.com (E.d.S.P.); gustavo.fontanari@ufra.edu.br (G.G.F.); 4 Faculdade de Nutrição (FANUT), Instituto de Saúde, Universidade Federal do Pará (UFPA), Rua Augusto Corrêa, Belém 66075-900, Pará, Brazil; 5 Instituto de Saúde e Produção Animal (ISPA), Universidade Federal Rural da Amazônia (UFRA), Belém 66077-830, Pará, Brazil; 6 Laboratório de Ecologia e Evolução, Instituto Butantan, São Paulo 05503-900, São Paulo, Brazil

**Keywords:** andiroba residue, central composite rotational design, bioactive compounds, non-conventional extraction technologies, biological in vitro activities

## Abstract

Methods for extracting bioactive compounds are widely studied, as the global trend moves toward more efficient and eco-friendly methods. This study aimed to optimize and compare the extraction of phenolic compounds from the residual biomass of andiroba (*Carapa guianensis*) using the conventional method with methanol as the solvent, and a green chemistry method via ultrasound using ethanol as the solvent. The optimization of the extraction variables (time, mass/volume ratio, and ethanol concentration) was performed using a Central Composite Rotatable Design (CCRD 2^3^) combined with the desirability function. The established optimal conditions were an extraction time of 26.78 min, a mass/volume ratio of 86.46 mg/mL, and an ethanol concentration of 32.5%. The conventional method achieved a higher yield of total phenolic compounds (TPC) (109.05 mg GAE/mL) and total flavonoid compounds (TFC) (38.18 mg GAE/mL) compared to the ultrasound-assisted method (UAE) (82.64 and 26.27 mg GAE/mL, respectively). LC-MS analysis revealed a diversity of extracted bioactive molecules. Both methodologies yielded extracts with good in vitro antioxidant activity (DPPH and ABTS). In the antimicrobial assay, the extracts demonstrated an unprecedented bacteriostatic effect for this residue, inhibiting the growth of the Gram-positive bacterium *Staphylococcus aureus* at concentrations ranging from 43.83% to 52.67%, with no activity against Gram-negative bacteria. The optimization demonstrated that the industrial residue of andiroba still contains a significant concentration of bioactive compounds. These findings confirm the bioeconomic potential of this by-product for the formulation of high-value-added bio-inputs, with promising applications in the development of smart packaging or bioactive food coatings.

## 1. Introduction

*C. guianensis*, an Amazonian seed, yields an oil traditionally employed in folk medicine primarily for its analgesic and anti-inflammatory benefits. Beyond these traditional uses, the oil demonstrates a broad spectrum of biological properties, including insecticidal, repellent, acaricidal, vermicidal, and antimalarial (against *Plasmodium falciparum*) activities, along with anti-edematogenic and anti-allergic effects, all while presenting a low teratogenic potential [1].

*C. guianensis* belongs to the Meliaceae family and is commonly known as andiroba, a word originating from Tupi-Guarani meaning “bitter taste” [2]. Andiroba oil is highly valued by industry due to its lipid profile of palmitic acid (20.2%), oleic acid (45.5%), linoleic acid (8.3%), linolenic acid (0.8%) and stearic acid (5.3%) [3].

The processing of oilseeds in the industries that benefit them naturally generates waste that is often disposed of irregularly or accumulates in nature. Normally, these are used for energy generation, fertilizers or animal feed production [4,5].

The industrial sector generates significant quantities of biomass. Improper disposal of these materials leads to serious environmental and phytosanitary problems, while also wasting valuable resources that could otherwise be recovered [6]. Andiroba residual biomass is a valuable source of oil. This oil consists mostly of saponifiable fatty acids (such as oleic, palmitic, stearic, and linoleic acids), alongside unsaponifiable matter containing steroids, triterpenes, and primarily limonoids [7].

In the residual biomass, it is still possible to find phenolic compounds, which are very abundant and form the basis for several biological activities of plants [8]. Some compounds, even being minor in the composition of foods, have significantly beneficial effects on human health [9]. One or more hydroxyl groups on at least one benzene ring define phenolic substances. Special properties like antioxidant, anti-carcinogenic, anti-inflammatory, anti-viral, anti-microbial, hypomeliative, and hypoglycemic power are conferred by their chemical structures [10].

The amount and quality of phenolic chemicals recovered from plant matrices are greatly influenced by the extraction techniques used. All of the many approaches currently in use have good extraction quality; nevertheless, their application is limited by a number of issues, including the use of hazardous solvents, high temperatures, polarity, and solubility [8]. Thus, ethanol, a polar solvent that can be obtained sustainably and is permitted for use by the Food and Drug Administration (FDA), has high efficacy in the extraction of phenolic compounds and is usually used with added water [11].

In contrast to traditional techniques that depend on extended heating and run the danger of breaking down target molecules [4], UAE provides a quicker, more effective, and more affordable option [12]. The UAE mechanism relies on acoustic cavitation to rupture plant cell walls, significantly enhancing solvent penetration and mass transfer while operating at lower, controlled temperatures to protect thermosensitive compounds [13]. Furthermore, combining this acoustic technology with aqueous ethanol, a green solvent, further optimizes the sustainable and safe recovery of phenolic compounds [11].

Design of Experiments (DOE) optimizes processes and minimizes costs by systematically evaluating variable interactions through fewer empirical trials. As a specific DOE method, the five-level Central Composite Rotational Design (CCRD) incorporates axial and center points to ensure model orthogonality and rotatability, enabling a precise exploration of the experimental domain [14,15].

Although *C. guianensis* has a historically established application in the cosmetics industry and traditional medicine, it shows immense potential for exploration as a bio-input in the food industry. The scientific literature has demonstrated that the bioactive compounds present in its extracts and oils (such as limonoids and fatty acids) have significant antimicrobial activity. Several studies demonstrate the effectiveness of these compounds in inhibiting microorganisms of great interest for food safety and preservation, such as pathogens that cause foodborne illnesses, like *S. aureus* [16].

The residues generated after extraction retain a rich composition of bioactive compounds that can be utilized, thereby adding value to the waste material. The extracted limonoids exhibit antimalarial activity in both in vitro and in vivo models and show no toxicity to human cells [17]. This bioactivity makes *C. guianensis* an interesting natural alternative for the development of biopreservatives or for the formulation of active packaging, adding biotechnological and sustainable value to the food production chain.

To maximize the extraction of the compounds, this study uses a full star experimental design with response surface methodology (RSM) as a tool to optimize the extraction of phenolic compounds by UAE from andiroba. The effects of the following variables were assessed: extraction time (min), solid–liquid ratio (% *w*/*v*), and ethanol concentration (% *v*/*v*).

## 2. Materials and Methods

### 2.1. Reagents and Biological Materials

Analytical- or HPLC-grade chemicals and reagents were utilized: Absolute ethanol (99%, Dinâmica^®^ Ltda (São Paulo, SP, Brazil)) was utilized as the solvent for extract production. For the quantification of bioactive compounds, Folin–Ciocalteu reagent (2 N, Dinâmica^®^ (São Paulo, SP, Brazil)), calcium carbonate (98%, Neon (Suzano, SP, Brazil)), anhydrous aluminum chloride (99.99%, Sigma-Aldrich, St. Louis, MO, USA), gallic acid monohydrate (98%, Sigma-Aldrich, St. Louis, MO, USA), and quercetin (95%, Sigma-Aldrich, St. Louis, MO, USA) were employed. Antioxidant assays were conducted using 2,2′-azino-bis (3-ethylbenzothiazoline-6-sulfonic acid) (ABTS, 98%, ACS Científica (São Paulo, SP, Brazil)), 2,2-diphenyl-1-picrylhydrazyl (DPPH, 97%, Sigma-Aldrich, St. Louis, MO, USA), and Trolox (97%, Sigma-Aldrich, St. Louis, MO, USA). Antimicrobial evaluations required dimethyl sulfoxide (DMSO, 99%, Dinâmica^®^ (São Paulo, SP, Brazil)), chloramphenicol (99%, Êxodo Científica (São Paulo, SP, Brazil)), Mueller–Hinton agar and broth (Kasvi^®^ (Pinhais, PR, Brazil)), and resazurin (INLAB^®^ (São Paulo, SP, Brazil)). Finally, chromatographic analyses were performed using ultrapure water, formic acid, and HPLC-grade acetonitrile, all supplied by Sigma-Aldrich (St. Louis, MO, USA).

### 2.2. Equipment

The equipment used in this research included a Sigma brand centrifuge, model 3K30, acquired in the city of Osterode am Harz, Germany. For absorbance readings, a Thermo Scientific Multiskan Sky microplate spectrophotometer (reference Go-SN-1530-8001397), from Vantaa, Finland, was used. The lyophilization process of the extract was carried out in a LIOBRAS brand freeze dryer, model M-L108, acquired in São Carlos, SP, Brazil. More complex analyses required a set of equipment from Shimadzu Corporation, all acquired in Kyoto, Japan: an ESI-IT-TOF mass spectrometer, a UFLC Prominence 20A liquid chromatography system, and an SPD-M20A diode array detector. The following were used for FTIR analysis: a freeze dryer (LIOBRAS, model M-L108, São Carlos, SP, Brazil) and a Fourier Transform Infrared Spectrometer (FTIR) (Thermo Scientific, model Nicolet 6700 FT-IR, Madison, WI, USA). Antimicrobial activity analyses involved incubation in a DeLeo^®^ brand incubator, model TLK 48, from Porto Alegre, RS, Brazil, and bacterial growth readings were taken using an OLABO^®^ spectrophotometer, model BK-EL10C, originating from Jinan, Shandong, China.

### 2.3. Sample Collection

The *C. guianensis* residual biomass used in this research was a byproduct of cold-pressing oil extraction, supplied by AmazonOil S.A. (Ananindeua, PA, Brazil). Research utilizing this botanical material is authorized by SisGen (registration no. A2D84C7). Initially milled at the supplier’s facility using a ball mill, the pressed cake was subsequently transported to the Animal Nutrition Laboratory at Universidade Federal Rural da Amazônia. There, the material was passed through a 60-mesh sieve, portioned into aliquots of roughly 300 g, vacuum-sealed in polyethylene bags, and stored at ±5 °C prior to analytical procedures.

### 2.4. Central Composite Rotational Design (CCRD) for Total Phenolic Content Extraction

To optimize the TPC extraction conditions, a CCRD 2^3^ was developed, totaling 17 trials, as presented in Table 1. The independent variables evaluated were temperature (X_1_), solid:liquid ratio (X_2_), and reagent concentration (Ethanol) (X_3_). The design includes 8 factorial points (k = 3), 6 axial points for quadratic evaluation, and 3 repetitions at the central point for estimating experimental error, considering pure error. The dependent variables for this study were defined as Y_1_: TFC and Y_2_: TPC expressed in milligrams of quercetin and gallic acid per milliliter (mg GAE/mL) of sample, respectively; their determinations are detailed in Section 2.5 of this manuscript.

To define the ranges for the experimental design, this study followed our previous studies in the references [5,18].

To prevent systematic biases caused by uncontrolled environmental or equipment variations, all extraction trials were executed in a randomized sequence. For subsequent mathematical modeling and response surface generation, the experimental data were reorganized into standard order. In this study, a 95% confidence interval (*p* < 0.05) was used for significance of the regression coefficients.

UAE (40 kHz, 100 W, 35 °C) of the sieved biomass (<150 mesh) was performed in Schott-type flasks using continuous ultrasonic pulsing, while varying the hydroalcoholic concentration and time according to the experimental design (Table 1). The liquid fraction was recovered by centrifugation (10,000× *g*, 20 min), rotary-evaporated to remove the solvent, and kept at −18 °C in amber bottles until further characterization.

#### 2.4.1. Processing of CCRD Data

Statistical analysis was performed using regression modeling in RStudio software version 4.4.2 [19], then a second-order polynomial equation, represented by Equation (1), was fitted to the experimental data in order to get the regression coefficients [20,21].

(1) $$Y=\beta_{0}+\sum_{i=1}^{k}\beta_{i}x_{i}+\sum_{i=1}^{k}\beta_{ii}\beta_{i}^{2}+\sum_{i<j}\beta_{ij}+x_{i}x_{j}+\epsilon$$

where Y is the estimated response; β_0_ is the constant (intercept); βi is the linear coefficient; β_ii_ is the quadratic coefficient; β_ij_ is the interaction coefficient; xi and x_j_ are coded independent variables; and *ϵ* is the experimental or residual error, or the difference between the observed value and the value predicted by the model.

A Pareto chart was employed to identify the significant linear, quadratic, and interaction effects at a 95% confidence level (*p* < 0.05). Non-significant terms were excluded to refine the predictive models. The mathematical adequacy of the adjusted models was then validated through Analysis of Variance (ANOVA), utilizing F-tests to confirm both the significance of the regression and a non-significant lack of fit. For a model to be considered significant and predictive, the F-value calculated for the regression must be greater than the regression F-value obtained from the standard table; furthermore, the F-value calculated for lack of fit must be less than the tabulated F-value.

#### 2.4.2. Obtaining the Optimized Extract

Three-dimensional response surface plots were created after the model was validated to show how the independent variables interacted with the extraction process. Using Harrington’s desirability function, all examined parameters were simultaneously optimized [22]. This multi-response optimization approach transforms the individual responses into a single overall desirability index (ranging from 0 to 1), allowing for the determination of the optimal operational conditions that maximize the recovery of bioactive compounds.

According to Harrington’s methodology [22], the obtained desirability values (*d*) are interpreted based on a specific scale. The responses are categorized as ‘very acceptable’ for values ranging from 0.80 to 1.00, and ‘acceptable’ for the 0.63 to 0.79 range. Values between 0.37 and 0.62 are considered ‘satisfactory’. Conversely, desirability indices below 0.37 are deemed inadequate, being classified as ‘not acceptable’ (0.20–0.36) and ‘very not acceptable’ (0.0–0.19).

#### 2.4.3. Model Validation

In order to determine the Mean Absolute Percentage Error (MAPE) using Equation (2), the conditions for this model were evaluated in a lab. MAPE values of up to 10% were thought to be ideal for this study’s model validation.

(2) $$MAPE=\frac{1}{n}\sum_{i=1}^{n}\left|y_{i}-p_{i}\right|.100$$

where *n* is the total number of samples; y_i_ is the experimental value for sample i; and p_i_ is the predicted value for sample i.

### 2.5. Conventional Extraction

To establish a baseline for maximum phenolic recovery and compare it with ethanol UAE extraction, methanolic extracts were prepared. The same number of grams found in the optimal condition of *C. guianensis* was exhaustively macerated in 50 mL of 90% (*v*/*v*) methanol. The mixtures were then incubated in the dark (covered with aluminum foil) at a constant 25 °C for 24 h. Following centrifugation to remove solid debris, the solvent was evaporated, and the sample was stored under the same conditions as the experiment was carried out. This sample was subjected to Bioactive and antioxidant analysis in order to make comparisons between the extraction methods [23].

### 2.6. Bioactive Compounds

#### 2.6.1. Total Phenolic Content

TPC was analyzed for all 17 trials using the methodology of Singleton et al. [24], and it was adapted for a microplate spectrophotometer. The absorbance at 765 nm was measured. Milligrams of gallic acid equivalents per milliliter (mg GAE/mL) were used to express the results, which were based on a gallic acid standard curve (5–200 mg/L; y = 0.0069x + 0.93, R^2^ = 0.9899).

#### 2.6.2. Total Flavonoids Content

Using a modified methodology [25], TFC was only quantified under ideal experimental conditions. The process involved combining 115 µL of the diluted extract with 115 µL of a 2% aluminum chloride (AlCl_3_) solution in a microplate. The absorbance was measured at 425 nm after a brief 30 s agitation and ten minutes of rest. A quercetin standard curve (y = 0.0167x − 0.0188, R^2^ = 0.9911) was used to quantify the concentration, and the results were reported in milligrams of quercetin equivalent per milliliter of concentrated extract (mg QE/mL).

### 2.7. Analyses Performed on the Extract Under Optimized Conditions

#### 2.7.1. Fourier Transform Infrared Spectroscopy (FTIR)

For spectral acquisition, the extract was initially lyophilized and homogenized with potassium bromide (KBr). The evaluation of functional groups was performed by FTIR, with measurements in transmittance mode and an incidence angle of 45°. Data collection occurred in the range of 4000 to 650 cm^−1^, with a resolution of 4 cm^−1^ and an average of 32 scans per minute, the process being managed by the OMNIC v. 9.2 software.

#### 2.7.2. LC-MS Analysis

The optimized extract (which had previously been filtered through 0.22 µm Millex filters) was analyzed by LC-MS using a Shimadzu system that included an ESI-IT-TOF mass spectrometer (Kyoto, Japan) and a UFLC connected to a PDA detector. Using a 35 min linear gradient (0 to 40%) of mobile phases of water and acetonitrile acidified with formic acid, chromatographic separation was carried out on a Kinetex C18 column at a flow rate of 0.2 mL/min. The last detection collected data in the *m*/*z* range of 300–1000 while operating in ESI mode.

#### 2.7.3. Antioxidant Activity by ABTS and DPPH

The extracts’ antioxidant activity was assessed in vitro by ABTS [26] and DPPH [27] microplate spectrophotometer-compatible colorimetric tests. The results were expressed in µM of TE/mL of concentrated material and measured using the Trolox standard in both procedures. The linear equation y = −0.0002x + 0.7055 (R^2^ = 0.9902) served as the basis for quantification in the ABTS assay, whereas the equation y = −0.0026x + 0.7873 (R^2^ = 0.9987) guided the DPPH assay.

#### 2.7.4. Antimicrobial Activity

The broth microdilution method was used to assess antimicrobial activity in accordance with CLSI standards [28]. The extracts were previously dissolved in DMSO (10%) and subjected to serial dilutions to achieve final concentrations ranging from 20 to 0.15 mg/mL. ATCC strains of *Escherichia coli* (ATCC 25922), *S. aureus* (ATCC 25923), and *Pseudomonas aeruginosa* (ATCC 27853) were used in the assay; the inoculum was standardized to attain roughly 5 × 10^1^ CFU/mL in the wells. The plates containing the samples, a solvent control, and a positive control (1 mg/mL of chloramphenicol) were incubated for 24 h at 35 ± 2 °C. Following incubation, absorbance measurements at 625 nm using a spectrophotometer (ELISA Microplate reader, BK-EL10A, OLABO, Jinan, China) were used to determine the percentage of bacterial growth suppression (Equation (3)).

(3) $$I\left(\%\right)=\frac{\left({ABS}_{control}-({ABS}_{sample}-{ABS}_{blank})\right)}{{ABS}_{control}}\;\times \;100$$

where ABS_sample_ is the absorbance of the extract with inoculum after 24 h incubation, ABS_blank_ is the absorbance of the extract without inoculum, and ABS_control_ is the absorbance of the inoculum without extract.

Using the resazurin assay (0.01% (*m*/*v*)), preliminary antibacterial screening evaluated the percentage of bacterial growth inhibition and expressed the results in relation to the control at doses that demonstrated discernible inhibition. The lowest extract concentration that can totally stop this indicator’s color shift is known as the Minimum Inhibitory Concentration (MIC). The Minimum Bactericidal Concentration (MBC) was calculated by culturing 10 µL aliquots from wells without visible growth on Mueller–Hinton agar plates and incubating them at 35 ± 2 °C for 24 h. The MBC was defined as the lowest concentration that did not cause colonies to develop visibly [29].

### 2.8. Statistical Analysis

The results obtained were presented as mean and standard deviation. To compare the bioactive compounds and antioxidant activity between UAE (ethanol) (at optimal conditions) and exhaustive maceration (70% methanol), Student’s *t*-test was applied. Prior to parametric tests, data normality and homogeneity of variances were assessed using the Shapiro–Wilk test. Specifically for antimicrobial analyses, one-way ANOVA was used to compare continuous variables among more than three samples in relation to the concentrations studied. If a significant difference was found in the ANOVA, Tukey’s post hoc test was used for multiple comparisons, according to the result of the Shapiro–Wilk normality test. All analyses considered a 95% confidence level (*p* < 0.05) and were performed using RStudio software version 4.4.2.

## 3. Results

### 3.1. Experimental Design and Optimization

**Figure 1:**
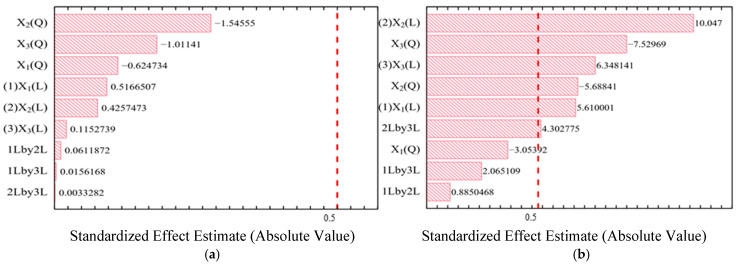
Pareto chart, showing the influence of the study variables (X_1_, X_2_ and X_3_, and their interactions) on responses Y_1_ (**a**) and Y_2_ (**b**).

The Pareto chart shows the variables and their influence Figure 1.

The statistical significance of the independent variables, extraction time (X_1_), solid–liquid ratio (X_2_), and solvent concentration (X_3_), on the evaluated responses was determined through the analysis of standardized effects Pareto charts, adopting a confidence level of 95% (*p* < 0.05).

Non-significant effects (*p* > 0.05) were ignored, and an ANOVA was performed, as shown in Table 2.

The regression model proved to be highly significant (*p* < 0.05), evidenced by the calculated F (7.25) being higher than the tabulated F (3.22). In parallel, the lack of fit test was not significant, presenting a calculated F (6.99) strictly lower than the tabulated limit (19.37). This behavior proves that the second-order polynomial equation satisfactorily fits the experimental data, and the observed deviations result from pure error in the analytical process, and not from mathematical inconsistencies in the model. The coefficient of determination (R^2^ = 0.81) indicates that the model can explain 81% of the variance in extraction yields.

**Figure 2:**
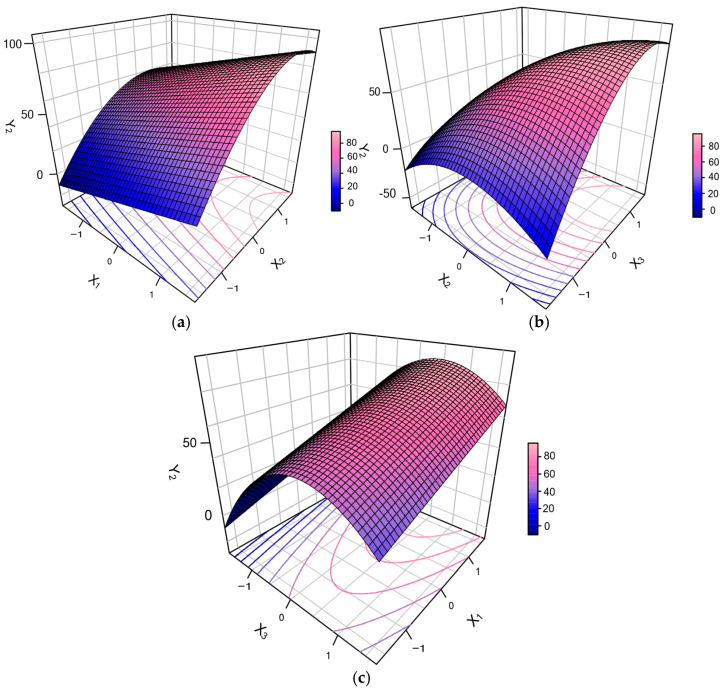
Response surface: (**a**) X_1_ and X_2_; (**b**) X_2_ and X_3_; and (**c**) X_3_ and X_1_. X_1_: time (min); X_2_: ratio mass/volume; X_3_: solvent concentration (% *v*/*v*).

After the model satisfied the F-tests, the response surfaces were generated, which can be seen in Figure 2.

Analyzing the surfaces, it is observed that time (X_1_) acts linearly and positively continuously in Figure 2a,c, showing that prolonging the operation constantly favors extraction. In contrast, the mass/volume ratio (X_2_) and the solvent concentration (X_3_) exhibit strong quadratic behaviors, forming parabolic curves that prove the existence of ideal limits. In Figure 2b, which illustrates the intense synergy between X_2_ and X_3_, it reveals the formation of a clear peak (dark pink region) where the maximum yield of phenolic compounds is only achieved by simultaneously balancing the amount of matrix and the solvent strength, indicating that any significant excess or deficiency in either of these two parameters causes the system’s efficiency to drop drastically.

**Figure 3:**
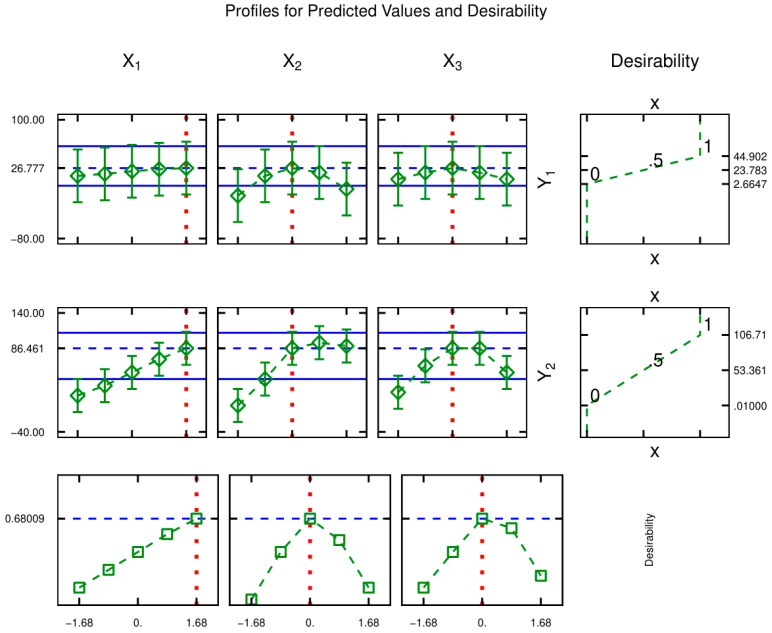
Desirability for optimizing the extraction of bioactive compounds from *C. guianensis* biomass.

To determine the ideal extraction conditions that simultaneously maximize the evaluated responses (Y_1_ and Y_2_), the Desirability Function tool was used (Figure 3). The analysis of the generated profiles indicates that the overall process optimization reached a desirability index of approximately 0.68 (considered “acceptable” by Harrington’s methodology [22]).

As illustrated by the dashed red lines (Figure 3), the optimal point for operation requires that the extraction time (X_1_) be taken to its maximum tested level (+1.68). Conversely, the mass/volume ratio (X_2_) and the solvent concentration (X_3_) must be strictly maintained at their central points (0). This result corroborates the analysis of the response surfaces, confirming the positive linear behavior for time and the strong quadratic saturation effect for the matrix ratio and solvent strength. Under these optimized operating conditions, the mathematical model predicts maximum values around 26.78 for Y1 and 86.46 mg/mL for the extraction of TPC (Y_2_).

It is important to highlight a particular aspect of the modeling: although the previous analysis of variance demonstrated that the variable Y_1_ did not obtain a statistically significant mathematical model or one with strong predictive character, it was intentionally maintained in the overall optimization. The inclusion of Y_1_ (TFC) in the desirability function is justified by the operational need to monitor the system holistically. In this context, the non-predictive response acts as a constraint variable or secondary control parameter. This ensures that the mathematical maximization of the primary and predictive variable (Y_2_: TPC) does not push the process towards extreme conditions that could nullify or severely compromise the extraction of Y_1_.

The results of the mathematical model validation are shown in Table 3.

To confirm the reliability of the operating conditions maximized by the desirability function, experimental validation tests were conducted at the optimal point established by the model. The practical results (Table 2) demonstrated excellent adherence to the mathematically predicted values.

### 3.2. Comparison Between Bioactive Compounds and Antioxidant Activity Using Conventional and Ultrasound Methods

**Figure 4:**
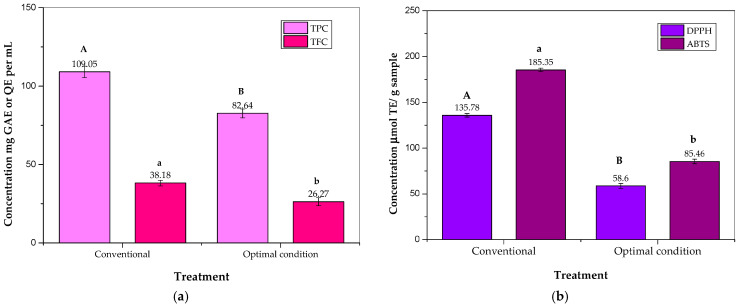
(**a**) TPC and TFC (**a**), and antioxidant activity determined by ABTS and DPPH assays (**b**) for extracts obtained under optimized conditions and conventional extraction techniques. Means with the same letters (uppercase and lowercase) in the same column indicate no significant difference according to Student’s *t*-test (*p* < 0.05).

Figure 4 presents graphs comparing the TPC for the extract under optimized conditions (Figure 4a) and for the antioxidant activities (Figure 4b) in comparison with extracts obtained by the conventional exhaustive maceration technique using methanol.

In Figure 4, the comparison between the conventional extraction process (methanol maceration) and the optimized eco-friendly alternative extraction (ultrasound-assisted ethanol extraction) demonstrated that the conventional protocol was statistically higher (*p* < 0.05) recovering of bioactive compounds and preserving antioxidant capacity. As observed in Figure 4a, there was a significant reduction in TPC and TFC in the optimized condition compared to the conventional method. While methanol extraction obtained 109.05 mg GAE/mL of TPC and 38.18 mg GAE/mL of TF, the optimized condition reached 82.64 and 26.27 mg/mL, respectively.

Although extraction methodologies involve multiple variables (solvent, time, and temperature), optimization results compared to the conventional approach indicate that, while the conventional method maximizes yield, optimized UAE offers a faster and more sustainable alternative that preserves a substantial fraction (approximately 75%) of the TPC.

### 3.3. Fourier Transform Infrared Spectroscopy (FTIR)

**Figure 5:**
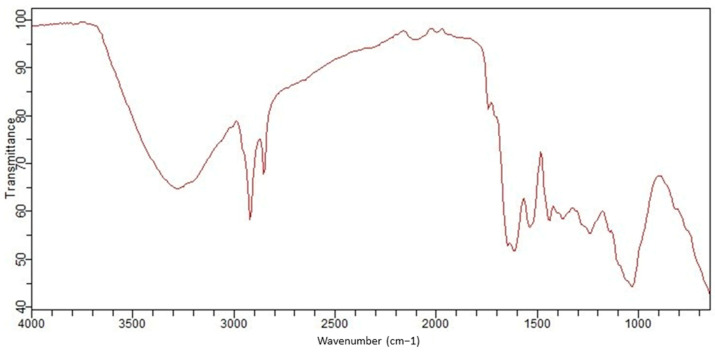
FTIR spectrum for the lyophilized extract of *C. guianensis.* Biomass extract under optimized UAE conditions.

Figure 5 shows the FTIR spectra obtained for fresh *C. guianensis* extract after freeze-drying.

The FTIR analysis of the lyophilized ethanolic extract of *C. guianensis* biomass revealed an absorption profile characteristic of complex mixtures, evidencing the extraction of both polar and non-polar compounds (Figure 5).

### 3.4. LC-MS Analysis

**Figure 6:**
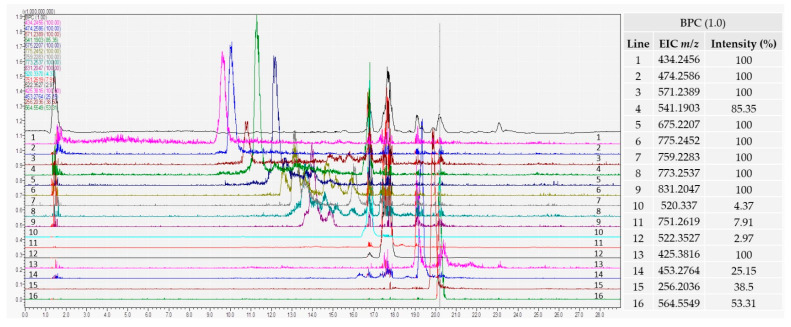
LC-MS profile of the *C. guianensis* extract, showing the total ion count (TIC) and chromatograms extracted for the main ions detected.

The chromatographic profile of the *C. guianensis* extract analysis is displayed in Figure 6.

The chromatograms, which are placed in “base-shift” style, or shifted on the Y-axis, are to the right of the enlarged table containing the values of the extracted ions and their corresponding color codes for improved visualization. The molecules in the C. guianensis extract began their chromatographic elution at lower concentrations of acetonitrile (~25%, RT ~8 min), according to the organic solvent extraction steps. A more noticeable concentration of molecules eluting from ~40% solvent B (Acetonitrile) was obtained, or about 17 min in the retention time. The molecular weights of all the ions mentioned match those of phytophenols, and they are all mono-charged.

The metabolic profiling of the *Carapa guianensis* extract by high-resolution mass spectrometry (LC-HRMS) revealed a complex phytochemical matrix characteristic of the *Meliaceae* family (Table 4). The putative annotation of the molecules with 100% relative abundance.

### 3.5. Antimicrobial Activity

The ethanolic extract of *C. guianensis* showed selective and bacteriostatic antimicrobial activity. Although it did not reach the MIC or MBC at the tested concentrations (0.16 to 1.75 mg/mL), it inhibited the growth of *S. aureus* (Gram-positive) by more than 50%. The absence of effect against Gram-negative strains (*E. coli* and *P. aeruginosa*) is justified by the complexity of their outer membrane, rich in lipopolysaccharides, which acts as a hydrophobic barrier to the penetration of phytophenols. Table 5 presents the results for the inhibition of *S. aureus* growth only at different concentrations of *C. guianensis* extracts.

## 4. Discussion

### 4.1. Experimental Design and Optimization

Pareto for FT (Y_1_) (Figure 1a) showed that none of the linear, quadratic, or interaction terms exceeded the significance line. This indicates that, within the experimental ranges delimited by the CCRD, the changes in operational parameters did not exert a statistically significant influence on this response variable.

On the other hand, the behavior observed for TPC (Y_2_) (Figure 1b) was different, showing a strong dependence on extraction conditions. The linear term of the solid–liquid ratio (X_2_) showed the largest positive standardized effect (10.047), showing that increasing the proportion of solid in relation to the solvent significantly favors the response. The second most impactful factor was the quadratic term of solvent concentration (X_3_), which showed a negative effect of −7.529. The significance of this negative quadratic term is of great importance, as it indicates a curvature in the response surface with concavity facing downwards, confirming the existence of a point of maximum extraction efficiency in the central region of the design.

Furthermore, the extraction time (X_1_), the linear influence of solvent concentration (X_3_), the quadratic term of the solid–liquid ratio (X_2_), and the interaction between the solid–liquid ratio and solvent concentration all demonstrated statistical significance (*p* < 0.05). These findings demonstrate the intricacy of the mass transfer process and the necessity of simultaneously optimizing the parameters to maximize the Y_2_ response.

The extraction profile for TPC aligns with other Amazonian residues, such as *Astrocaryum. murumuru* [5] and *P. macroloba* [4]. While the oil has well-established applications in the pharmaceutical and cosmetic industries, adding value to its residual cake closes the economic cycle and promotes the integral use of this raw material [35]. For instance, Sodré Souza et al. [35] evaluated the utilization of andiroba waste subjected to an alkaline pre-treatment to maximize the recovery of fermentable sugars. Through response surface methodology, their optimization model presented a coefficient of determination ($R^2$) of 0.95, establishing the optimal conditions at 100 min, 4% NaOH (*w*/*v*), and 120 °C, which allowed a saccharification yield of approximately 47.9%. Similarly to their approach, the use of a Central Composite Rotational Design (CCRD) in the present study proved to be a powerful tool to model and establish the ideal parameters for capitalizing on this food processing by-product, shifting its status from an industrial waste to a valuable bio-input.

The negative quadratic effect of the solvent (−7.529) and the high significance of the solid–liquid ratio confirm that hydroalcoholic mixtures (intermediate polarity) and strong concentration gradients are important to maximize cell diffusion and overcome mass transfer limitations.

Conversely, while [36] found ultrasound time and polarity to significantly affect flavonoid extraction in bee pollen, no variables were significant for FTC in this study. This suggests the *C. guianensis* matrix reaches a rapid solubilization plateau independent of the CCRD operational ranges. Furthermore, the solid–liquid ratio was the primary impact factor for TPC here (10.047), diverging from the literature that typically emphasizes solvent concentration or sonication time.

Regarding ANOVA (Table 2), the analysis of the coefficients of the mathematical equation reveals the magnitude of the influence of each operational parameter. The linear terms for time (X_1_), mass/volume ratio (X_2_), and solvent concentration (X_3_) showed positive coefficients (+10.71, +19.20, and +12.13, respectively), indicating that the initial increase in these variables favors the mass transfer of bioactive compounds to the extract, with the solid–liquid ratio being the main driving force of the system.

On the other hand, the presence of significant negative quadratic coefficients for the mass/volume ratio (−10.08X_2_^2^) and solvent concentration (−13.95.X_3_^2^) describes parabolic behavior on the response surface (Figure 2a–c), confirming the existence of an optimal saturation point for yield. Additionally, the positive interactive term (+10.74.X_2_.X_3_) shows a strong synergistic response between the solid matrix ratio and the solvation power of the extractor, demonstrating that the maximum efficiency of the process depends on the simultaneous adjustment of these two variables.

Regarding the desirability tool (Figure 3) on the extraction of TFC (Y_1_), the experimental value obtained was 26.27 mg/mL, very close to the prediction of 26.78 mg/mL, resulting in a MAPE of only 1.90% (Table 3). This low deviation reinforces the suitability of Y_1_ as a reliable constraint variable in the optimization. Similarly, the extraction of TPC (Y_2_), the primary variable of the study, achieved an experimental yield of 82.64 mg/mL, compared to the predicted 86.46 mg/mL. The MAPE of 4.60% for this response is considered highly satisfactory, especially since it involves the extraction of bioactive compounds in a complex lignocellulosic matrix. The small magnitude of the percentage errors (both less than 5%) (Table 3) proves that the regression model and desirability function applied are robust, reproducible, and suitable for predicting and optimizing the leaching process in the system studied.

### 4.2. Comparison Between Bioactive Compounds and Antioxidant Activity Using Conventional and Ultrasound Methods

In Figure 4, a similar pattern was reported by [37]. When comparing microwave-assisted extraction methods, ultrasound, and a combination of both in the phenolic profile of jackfruit pulp, the authors found that conventional extraction outperformed ethanolic extraction, obtaining 1.64 mg GAE/mL (methanol) versus 1.16 mg GAE/mL (ethanol).

Regarding *C. guianensis* biomass, the TPC and TFC values were significantly higher than those reported by [5], who found 0.248 mg GAE/mL and 0.01 mg QE/mL. It should be noted, however, that the authors expressed the results in relation to the extract mass, differing from the present study, which uses the sample mass as the basis for calculation.

Following the trend of bioactive compounds, antioxidant activity was substantially higher in the conventional method. The values for DPPH and ABTS were 135.78 and 185.35 µmol TE/g, respectively, while the optimized condition showed only 58.6 and 85.48 µmol TE/g.

The literature frequently reports a positive linear correlation between TPC/TFC levels and free radical scavenging capacity, since antioxidant activity tends to increase proportionally to the increase in phenolic compounds and the presence of free hydroxyls in the molecules [37]. Finally, when comparing the data with [38], who found ABTS values between 0.21 and 1.69 µmol TE/g and DPPH values reaching 4304.65 µmol TE/g for seeds of *Astrocaryum vulgare*, *Garcinia gardneriana* and *Bactris gasiapes*, it is observed that *C. guianensis* presents a lower prospect for the use of biomass in these specific applications.

These discrepancies should also be considered regarding ultrasound technology. In the study conducted by [39], pomegranate peels were subjected to a 25 min treatment period, 130 W of power, a 50% duty cycle, and a 50:1 liquid–solid ratio at a fixed frequency of 22 kHz. With a TPC of 21,521 mg GAE/mL of sample, these authors’ results were extremely similar to those of the current study. Enzyme extraction was also carried out by the same authors using the following parameters: 55 min, 50 °C, pH 5, and a 2% enzyme concentration. The greatest TPC of 30,608 mg GAE/mL was obtained by combining 25 min of ultrasonic treatment with 25 min of enzymatic treatment using this ultrasound-assisted method. This study demonstrated the high efficacy of ultrasound in phenolic compound extraction.

Conventional extraction using methanol remains more efficient in terms of total yield, as the type of solvent directly affects the solubility and extraction of target compounds based on the solvent’s polarity and affinity for the molecules [40].

Methanol is slightly more polar than ethanol, giving it a greater potential to more exhaustively extract the range of polar phenolic compounds from the sample. Furthermore, the conventional exhaustive extraction method exposes the material to a much longer contact time with the solvent, allowing for greater saturation of the extraction medium. Thus, although the ultrasound-assisted ethanol technique is greener, the conventional methanol-based methodology achieves a higher TPC extraction yield [41].

### 4.3. Fourier Transform Infrared Spectroscopy (FTIR)

By comparing the obtained FTIR spectrum with data from the literature, a comparison was made with pure *C. guianensis* oil, making it possible to identify the main functional groups present in the sample.

A broad and intense band centered at approximately 3350 cm^−1^ is observed. According to the literature on plant extracts, this region is strongly associated with stretching vibrations of hydroxyl groups (-OH), indicating the abundant presence of phenolic compounds, flavonoids, and polyols [39]. The intensity of this band contrasts with the typical profile of pure *C. guianensis* oil [40], in which triglycerides predominate and the -OH band is absent or very discreet, confirming that ethanolic extraction was efficient in concentrating the polar compounds of the biomass.

The spectra in Figure 5 display strong peaks at 2920 and 2850 cm^−1^. The (CH_2_) and (CH_3_) groups’ symmetric and antisymmetric stretching vibrational modes are represented by these signals. Despite the fact that research on extracts indicates that these signals might be connected to ethanol residues [39], in the context of *C. guianensis*, they perfectly coincide with the stretching of the acyl lipid chains [40]. This indicates that, even using ethanol as a solvent and lyophilizing the sample, the carried extract retained portions of fatty acids typical of the *C. guianensis* matrix.

In the literature consulted [40], pure *C. guianensis* oil showed a very intense band at 1745 cm^−1^, which may be associated with the (C=O) stretching of triglyceride esters. In the spectrum of the evaluated *C. guianensis* extract, this band at ~1745 cm^−1^ was practically imperceptible. However, a prominent band was observed in the 1610–1640 cm^−1^ region, attributed to the C=C stretching of aromatic rings and deformations linked to flavonoids and derivatives of hydroxycinnamic acids (such as caffeic acid), as observed in the reference that studied extracts of plant origin [16].

A broad band of very high intensity stands out in the sample’s spectrum, with a maximum peak near 1030–1050 cm^−1^. This band is strongly related to C–O [41] stretching vibrations of secondary alcohols, polyols (such as hydroxyflavonoids), and biomass carbohydrates [41].

The vibrational profile of polar enrichment observed for the ethanolic extract of *C. guianensis* finds a strong parallel in recent studies with other residual biomasses of Amazonian oilseeds. Ethanolic extraction followed by lyophilization of *A murumuru* resulted in analogous spectral profiles, marked by the intensification of bands in the regions of 3200–3600 cm^−1^ (O-H stretching), ∼1600–1500 cm^−1^ (aromatic C=C bonds) and ∼1050 cm^−1^ (C-O bonds) when compared to the raw matrix [5].

### 4.4. LC-MS Analysis

The LC-MS analysis (Figure 6) revealed the chemical complexity of the Carapa guianensis ethanolic extract, suggesting a composition that aligns with spectroscopic findings and the recent literature on Amazonian oilseed biomasses. In the absence of chromatographic standards, the putative annotation of metabolites (summarized in Table 4) provides a robust framework for interpreting this chemical profile. As shown in Table 4, the extract revealed a significant presence of complex polyphenolic compounds, including glycosylated flavonoids and condensed tannins and limonoids (tetranortriterpenoids), detected primarily as protonated ions ([M+H]^+^) ranging from m/z 425 to 831.

This elution profile and mass-to-charge ratios correlate with FTIR spectral features. Specifically, ions eluting at earlier retention times (8–13 min) suggest the presence of moderate-polarity compounds, justifying the intense absorption bands of hydroxyl groups (~3350 cm^−1^) and C-O bonds (~1050 cm^−1^) observed in the FTIR spectrum. Conversely, the high density of peaks at later retention times (16–21 min) points to the likely co-extraction of lipophilic molecules, consistent with aliphatic C-H stretching signals (2920 and 2850 cm^−1^), potentially related to residual fatty acids or less polar phenolic structures. The synergistic coexistence of these limonoids and polyphenols underpins the biological properties reported for andiroba. This prospective approach, correlating mass ranges with specific phytochemical classes, is consistent with methodologies recently validated for other Amazonian matrices, such as A. murumuru, demonstrating that the extraction process effectively concentrated a rich and diverse matrix of bioactive compounds [5].

### 4.5. Antimicrobial Analysis

Plant extracts high in phenolic compounds often exhibit this selective susceptibility pattern with respect to antibacterial action (Table 5). The structural complexity of Gram-negative bacteria, whose lipopolysaccharide and lipid-rich outer membranes function as a hydrophobic barrier that blocks the penetration of natural antimicrobial drugs, can be used to explain the lack of effectiveness against *E. coli* and *P. aeruginosa*. However, the absence of this selective outer barrier in the cell wall of Gram-positive bacteria like *S. aureus* makes them more vulnerable to interaction with the tannins and phytophenols found in the ethanolic extract of *C. guianensis.*

Although the extract did not demonstrate lethality (bactericidal effect), the partial inhibition observed against *S. aureus* indicates that the material acts through a predominantly bacteriostatic mechanism. This behavior corroborates recent studies with residues from other Amazonian oilseeds, such as *A. murumuru*, which also reported partial and concentration-dependent inhibition against *S. aureus*, without reaching the MIC or MBC under the tested conditions [5]. The ability to slow the growth of a clinically relevant pathogen, even without eradicating it, suggests that the bioactive compounds of *C. guianensis* interfere with the metabolism or cellular integrity of the bacteria.

Phytochemical compounds inhibit microbial proliferation by complexing with metal ions and proteins, without causing cell lysis. This effect is clinically significant, as it can facilitate the effective control of microorganisms and validates the use of andiroba in wound healing by protecting the host against severe infectious processes [16,42].

Considering the residual and sustainable origin of *C. guianensis* biomass, these preliminary results open perspectives for its application not as a primary antimicrobial, but as an adjuvant additive. The association of this extract, which has already demonstrated a rich chemical composition and probable antioxidant potential in cosmetic formulations, biofilms, or active packaging, can act synergistically to extend the shelf life of products and hinder bacterial proliferation, adding value to a byproduct of the Amazonian agro-industry.

It is worth mentioning that the scope of this study was limited to evaluating the in vitro biological activities and the technological potential of the extracts. To date, toxicological profiles and cytotoxicity data have not been established. Consequently, comprehensive safety assessments remain mandatory before these extracts can be safely proposed for practical applications, particularly in food-related products.

## 5. Conclusions

This study demonstrated the feasibility of extracting bioactive compounds from *C. guianensis* and its optimization using ultrasound-assisted therapy in conjunction with ethanol, significantly increasing the extraction of phenolic compounds. The experimental design (DCCR) proved to be reliable, feasible, and robust (R^2^ = 0.99 and adjusted R^2^ = 0.81), generating a significant and predictive model.

It is important to highlight that conventional extraction yielded higher absolute TPC and TFC values than the alternative extraction method (UAE). This result is attributed to the longer contact time and the superior solvating power of methanol, which allowed thermodynamic equilibrium to be reached. However, the alternative extraction method achieved approximately 75% of the total extraction capacity in considerably less time (26.78 min versus 24 h). By employing a sustainable, non-toxic solvent and requiring less processing time, UAE presents itself as an ecologically viable alternative for recovering bioactive compounds from this oilseed.

The optimization showed that FTC is rapidly solubilized and remains in a plateau region; however, the TPC is highly influenced by the solid–liquid ratio of the solvent concentration, which on the response surface exhibits parabolic behavior with well-defined minimum and maximum points.

LC-MS and FTIR analyses validated the effectiveness of the extraction methodology for concentrating bioactive compounds, such as phytophenols, thus preserving the characteristics of the matrix. Although the extract predominantly showed bacteriostatic activity against the tested strain, its bioactive composition suggests it is a promising candidate for future evaluation as an additive in cosmetic formulations or active food packaging. However, further comprehensive studies are strictly necessary, such as broader antimicrobial screening (in Gram-negative bacteria), cytotoxicity assays, and formulation compatibility tests, to establish the safety and functional viability of this bio-input. Therefore, obtaining the extracts, as demonstrated by predictive mathematical models, is a sustainable strategy for the integral use of *C. guianensis* biomass.

## Figures and Tables

**Table 1:** Real and coded variables of the experimental design of the CCRD matrix.

Variables	Levels				
Code	−α	−1	0	+1	+α
X_1_	0.34	15	37.5	60	75.34
X_2_	0.1	0.5	1.25	2	2.5
X_3_	0.01	0.5	32.5	60	78.75

Note: X_1_ = extraction time (min); X_2_ = solid–liquid ratio ((%) *m*/*v*); X_3_ = solvent concentration ((%) *v*/*v*).

**Table 2:** ANOVA table for Y_2_: TPC (mg/mL) response.

Effects on the Variable Y_2_					
Source of Variation	SQ	N° g.l.	MQ	F Calculated	Tabled F
Regression	12,541.6805	6	2090.2801	Regression	Regression
Residue	2883.1700	10	288.3170	7.25	3.22
Lack of fit	2783.56	8	347.945		
Pure error	99.61	2	49.807	Lack of fit	Regression
Total	15,424.85	16		6.99	19.37
R^2^	0.99				
Adjusted R^2^	0.81				
Mathematical model	Y_2_ = 68.45 + 10.71X_1_ + 19.20X_2_ − 10.08X_2_^2^ + 12.13X_3_ − 13.95X_3_^2^ + 10.74X_2_.X_3_				

G.L. for regression: 6:10 and *p*-value < 0.05; G.L. for lack of fit: 8:2 and *p*-value < 0.05. X_1_: time (min); X_2_: ratio mass/volume; X_3_: solvent concentration (% *v*/*v*).

**Table 3:** Validation of the mathematical model obtained by the desirability function.

Variable	Experimental Value (Y_i_)	Predicted Value (P_i_)	MAPE (%)
Y_1_	26.27 ± 2.50	26.78	1.90
Y_2_	82.64 ± 2.80	86.46	4.60

Y_1_: TFC (mg/mL); Y_2_: TPC (mg/mL).

**Table 4:** LC-HRMS chromatographic profiling and putative identification of metabolites.

Observed Ion (*m*/*z*)	Neutral Mass (*M*)	Putative Phytochemical Class	Reference
434.2456	433.2456	Triterpenoid	[30]
474.2586	473.2586	Tetranortriterpenoid	[31]
571.2389	570.2389	Limonoid	[32]
675.2207	674.2207	Complex polyphenol	[33]
759.2283	758.2283	Proanthocyanidin/Condensed tannin	[33]
775.2452	774.2452	Proanthocyanidin oligomer	[33]
831.2047	830.2047	Polyphenol	[33]
425.3816	424.3816	Triterpene	[34]

**Table 5:** Percentage of inhibition of the strains’ growth by the *C. Guianensis* optimized extract.

Sample	Concentration (mg/mL)	% Inhibition		
		*S. aureus* (ATCC 25923)	*E. coli* (ATCC 25922	*Pseudomonas* *aeruginosa* (ATCC 27853),
Chloramphenicol	1.00	100%	100%	100%
*C. guianensis* extracts	1.75	51.59 ± 11.80 ^a^	n.i	n.i
	0.63	43.83 ± 1.50 ^b^	n.i	n.i
	0.31	52.67 ± 5.24 ^a^	n.i	n.i
	0.16	44.17 ± 6.48 ^b^	n.i	n.i

Means with the same letters in the same column do not differ statistically from each other according to Tukey’s test (*p* < 0.05). ni: no inhibition.

## Data Availability

The original contributions presented in this study are included in the article. Further inquiries can be directed to the corresponding author.

## Abbreviations

The following abbreviations are used in this manuscript:

ABTS	2,2′-Azino-bis (3-ethylbenzothiazoline-6-sulfonic acid)
ANOVA	Analysis of Variance
DPPH	2,2-Diphenyl-1-picrylhydrazyl
ESI-IT-TOF	Electrospray Ionization Ion Trap Time-of-Flight
FTIR	Fourier Transform Infrared Spectroscopy
GAE	Gallic Acid Equivalent
LC-MS	Liquid Chromatography–Mass Spectrometry
MIC	Minimum Inhibitory Concentration
MBC	Minimum Bactericidal Concentration
PDA	Photodiode Array Detector
QE	Quercetin Equivalent
CCR	Central composite rotational (CCR) experimental design
RSM	Response Surface Methodology
SLR	Solid–Liquid Ratio
TAE	Tannic Acid Equivalent
TFC	Total Flavonoid Content
TIC	Total Ion Chromatogram
TPC	Total Phenolic Content
UAE	Ultrasound-Assisted Extraction
UFLC	Ultra-Fast Liquid Chromatography
